# Effects of Hyaluronic Acid on Three Different Cell Types of the Periodontium in a Novel Multi-Culture Cell Plate: An Exploratory Study

**DOI:** 10.3390/biom15010152

**Published:** 2025-01-20

**Authors:** Alessio Barbieri, Luciano Pitzurra, Bruno Loos, Ineke D. C. Jansen

**Affiliations:** Department of Periodontology, Academic Centre for Dentistry Amsterdam (ACTA), University of Amsterdam and Vrije Universiteit Amsterdam, 1081 LA Amsterdam, The Netherlands; l.pitzurra@acta.nl (L.P.); b.loos@acta.nl (B.L.)

**Keywords:** hyaluronic acid, periodontal cells, in vivo study, in vitro study

## Abstract

Hyaluronic acid (HA) has received considerable attention in the reconstruction of lost periodontal tissues. HA has been proposed to play a role in cell proliferation, differentiation, migration, and cell–matrix as well as cell–cell interactions. Although various studies have been conducted, further research is needed to expand our knowledge based on HA such as its effects on cell proliferation and osteogenic differentiation. The aim of this study is to assess, in single- and multi-culture plate models, the effect of HA on the proliferation, viability, and function of periodontal ligament fibroblasts, osteoblasts, and gingival epithelial cells. A novel multi-culture cell plate was chosen to simulate a cell–cell communication as close as possible to a real clinical condition in an in vitro setting. We found that HA exclusively enhanced epithelial cell proliferation, while intercellular communication stimulated the proliferation and osteogenic potential of the osteoblasts, independently from HA use. The proliferation and function of the periodontal ligament fibroblasts were not changed by HA or the cellular interplay. The use of multi-culture plates could represent a promising method to investigate and compare dental biomaterials in experiments mimicking an in vivo environment.

## 1. Introduction

Periodontitis is a chronic multifactorial inflammatory disease [1] of the tooth-supporting structures characterized by loss of connective tissue attachment and alveolar bone breakdown [2,3]. One of the goals of periodontal treatment is to promote the reconstruction of the lost periodontal tissues [4]. Periodontal regeneration is defined as the restoration of lost or diminished periodontal tissues including cementum, periodontal ligament, and alveolar bone (Glossary of Periodontal Terms, Chicago: American Academy of Periodontology, 2001). Over the past decades, the use of regenerative materials such as hyaluronic acid (HA) has become particularly popular in dentistry [5]. HA has been investigated in several surgical and non-surgical procedures for different dental and medical purposes [6].

HA is a major natural carbohydrate component of the extracellular matrix of vertebrates [7]. On the basis of the repeating disaccharides of glucuronic acid and N-acetylglucosamine composing HA chains [8], it is possible to discern low- and high-molecular-weight HA. In general, high-molecular-weight HA (900–104 kDa) seems to promote cell differentiation and improve or maintain cell–cell communication [9,10]. High-molecular-weight HA undergoes degradation during tissue repair to form low-molecular-weight HA (<10^3^ kDa) [11,12]. Low-molecular-weight HA exhibits pro-inflammatory and pro-angiogenic responses and stimulates proliferation and cell motility during tissue damage [11,12]. HA is not only a scaffold for cells or a filler for tissues, but it plays a primary role in processes such as cell motility, adhesion, proliferation, and cell–matrix as well as cell–cell interactions [13]. It can induce osteogenesis [14,15,16], and it seems to be osteoinductive [17,18]. The biological processes of HA are mediated by the attachment of HA to one of the three known HA cell surface receptors, namely CD44 (cluster of differentiation 44), RHAMM (receptor for hyaluronan-mediated motility), and ICAM-1 (intercellular adhesion molecule 1) [19].

For clinical use, HA can be chemically engineered to create a thicker gel. By transforming the liquid into a gel, polymer chains are cross-linked together to maintain the shape for a longer period of time and slow the process of disintegration [20]. The indications of HA in dentistry are very diverse and it is mainly used in periodontics and oral surgery [7]. In animal models, some authors showed histological and histomorphometric evidence of bone and periodontal regeneration with HA used alone [21] or in combination with other biomaterials [22]. Two randomized clinical trials (RCTs) in humans have confirmed additional benefits in terms of probing pocket depth reduction and clinical attachment level gain [23,24]. In the first RCT, HA application in periodontal infrabony defects determined a significant pocket depth reduction and clinical attachment level gain up to 24 months in periodontal infrabony defects compared to open flap debridement [23]. In the second RCT, similar clinical outcomes were reported when comparing HA to enamel matrix derivate in infrabony defects [24]. HA seems to promote keratinized gingival augmentation in mucogingival surgery [25] and to increase the effectiveness in achieving complete root coverage for single Miller class I/RT1 gingival recession sites [16].

In vitro studies showed promising results of HA on the viability of cells. The study by Fujioka-Kobayashi et al. [26] investigated the effects of HA on human periodontal ligament cells. The authors showed that HA can stimulate proliferation and promotes cell viability and early osteogenic differentiation in periodontal ligament cells. In the in vitro study on human oral fibroblasts by Asparuhova et al., HA was associated with higher migration and proliferation of oral fibroblasts [27]. In another study by Asparuhova et al., HA promoted the growth of osteoprogenitor cells and their stemness but also upregulated the expression of genes encoding bone matrix proteins [14]. Other studies demonstrated that HA can mediate earlier bone deposition of osteoblast cells by promoting gene expression of alkaline phosphatase activity, bone morphogenetic protein-2, and osteopontin [28,29,30]. Although many studies have been conducted, further investigations are needed to establish the precise mechanism of HA molecular weight and its concentration on cell proliferation and osteogenic differentiation [31].

Only single-culture plate in vitro studies have been performed to investigate the effects of HA on osteoblasts, periodontal ligament cells, and epithelium cells. We hypothesize that a multi-culture plate model could mimic the cell–cell interplay among different periodontal cell types and reproduce the cell–HA interaction closer to a real clinical situation compared to a single-culture plate model. This study model may expand our knowledge of the role of HA in periodontal regeneration procedures. Furthermore, we presume, in agreement with the literature [14,27,28], that HA will have a stimulatory effect on the proliferation of osteoblasts and periodontal ligament cells. The aim of this in vitro study is to assess, in single- and multi-culture plate models, the effect of HA on the proliferation, viability, and function of periodontal ligament fibroblasts, osteoblasts, and gingival epithelial cells.

## 2. Materials and Methods

### 2.1. Study Design and Cell Culture

**Figure 1:**
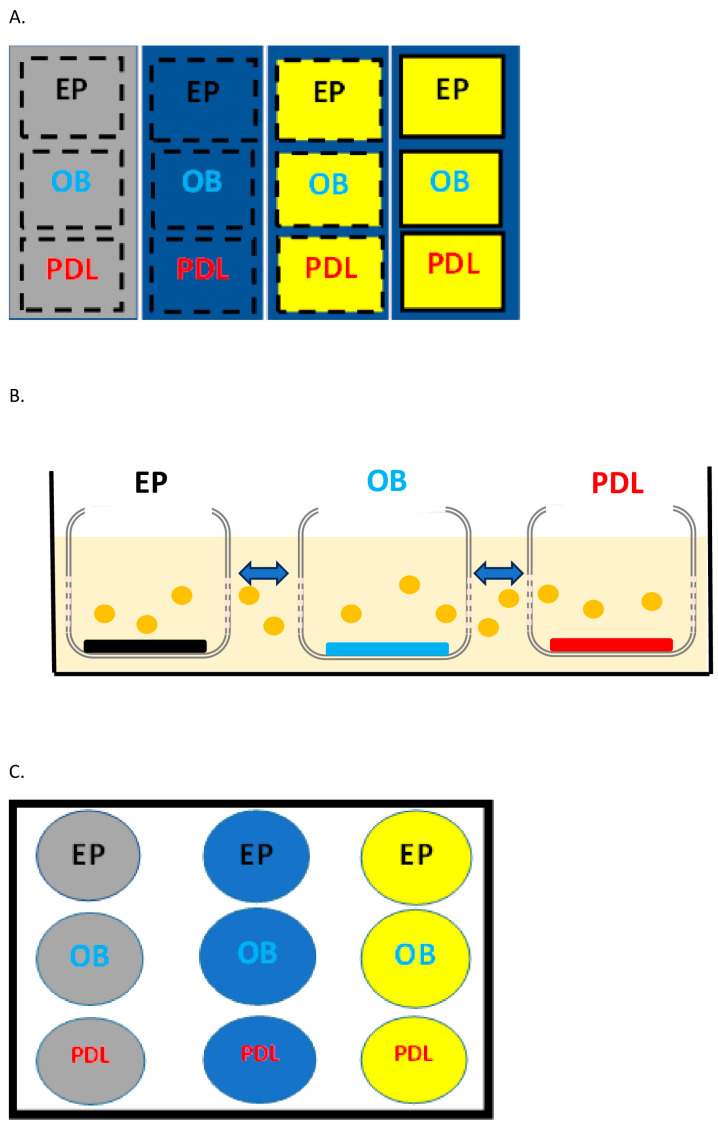
Control plate and experimental plate design. Graphical representation of the experimental plate (CELLBLOKS^®^) (**A**). EP: epithelial cell; OB: osteoblast; PDL: periodontal ligament fibroblast; grey color: medium without HA; blue color: medium with diluted HA; yellow color: medium with HA coated on the plate; dashed line: cell blocks with semi-permeable membrane; solid line: cell blocks without semi-permeable membrane. Experimental plate design and cell–cell communication through the semi-permeable membrane (**B**). Graphical representation of the control plate (**C**).

This in vitro study was approved by the ethical review committee (ETC) of the Academic Centre of Dentistry Amsterdam (ACTA) under the number ETC ID: 2021-99680. Periodontal ligament fibroblasts (PDLs) were retrieved from extracted wisdom teeth. PDLs were anonymized immediately after extraction for research use, according to the Dutch law. Osteoblasts (OBs) (cell line U2OS) (ATCC, Manassas, VA, USA) and gingival epithelial cells (EPs) (cell line CA922) (HSRRB, Osaka, Japan) were commercially obtained. Before the start of the experiment, each cell type was cultured in a medium composed of 50% Dulbecco’s Modified Eagle Medium (DMEM/F 12 GlutaMAX, Invitrogen Life Sciences, Carlsbad, CA, USA), 50% Minimum Essential Medium (MEM Alpha Medium, Invitrogen Life Sciences), 5% FCI (Fetal Clone I, HyClone Laboratories, Logan, UT, USA) and 1% PSF (antibiotics penicillin/streptomycin/amphotericin B, Sigma-Aldrich, St. Louis, MO, USA). The HA used in this experiment (hyaDENT BG, BioScience GmbH, Dümmer, Germany) was kindly provided by Regedent AG (Zurich, Switzerland). HyaDENT BG is a formulation containing cross-linked HA composed of 2 mg/mL sodium hyaluronate + 16 mg/mL cross-linked 1000 kDa HA monomers of butanediol diglycidyl ether. According to the manufacturer, the cross-linked formulation is engineered to slow the resorption rate of HA and to obtain a gel-type product that facilitates its clinical application. To simulate cell–cell communication, a special multi-culture cell plate (CELLBLOKS^®^, Revivocell©, Daresbury Laboratory, Daresbury, UK) was chosen as the experimental plate. CELLBLOKS^®^ is a “plug and play” co-culture technology which emulates an organ microenvironment in a standard in vitro setting. This experimental plate consists of four separated channels, and each channel contains three cell blocks in which the periodontal cells were cultured. The cell blocks can be inserted in the channels or removed and can be equipped with 1 μm pore size semi-permeable membranes. These membranes are designed to impede cell migration from a cell block to another one but to allow the medium to flow through the cell blocks, promoting a chemical interaction between different cell types (Figure 1A,B). The four channels are separated; therefore, there is no exchange of medium among the channels. For the experiments, 20.000 cells/mL of the three cell types was cultured in the CELLBLOKS^®^ and in a standard 12-well culture plate as a control (Greiner bio-one, Kremsmünster, Austria) (Figure 1C) without or with HA. Regarding the preparation of CELLBLOKS^®^, in the first channel, no HA was added in the medium. In the second channel, HA was diluted into the medium (1:100) with a final concentration of 180 μg/mL. The third channel was coated with HA. In this case, HA was diluted in a 1:10 proportion in sterile water, meaning that 100 µL of HA was mixed in 900 µL of sterile water; 100 µL of this preparation was added into the cell blocks of the third channel. These cell blocks were left for a few days (at least 3 days before the start of the experiment) until the water was completely evaporated and the remains of the HA were visible to the naked eye on the bottom of the blocks. The fourth channel was prepared in the same way as the third channel, but no cell interaction was simulated in this channel since the cell blocks were not equipped with the semi-permeable membranes. In order to completely cover the semi-permeable membranes, an extra amount of 4.5 mL medium was added into each channel. The preparation of the control plate wells followed the same procedure used for the experimental plates. The experiment was repeated at least three times.

### 2.2. Cell Proliferation and Viability

On day 3, the proliferation (viable cell/mL) and viability (%) of PDLs, OBs, and EPs of the experimental and control plates were assessed with a Muse^®^ Cell Analyzer (Merck KGaA, Darmstadt, Germany), according to the manufacturer’s protocol.

### 2.3. Quantitative Polymerase Chain Reaction (qPCR)

Gene expression was performed for BMP2 (bone morphogenetic protein 2), ALP (alkaline phosphatase), coll1A (collagen Type I alpha), CD44, TGF-β1 (transforming growth factor beta-1), ICAM-1, E-cad (E-cadherin), and housekeeping HPRT. An RNeasy Mini Kit (Qiagen, Hilden, Germany) was used to isolate RNA from PDLs, OBs, and EPs according to the manufacturer’s instructions. With a multilabel plate reader (The Synergy™ HT, BioTek Instruments, Winooski, VT, USA), the RNA concentration was measured. After that, the RNA was reverse-transcribed to cDNA with the first strand synthesis kit (Fermentas, Vilnius, Lithuania). The qPCR reactions were performed with a Lightcycler (Roche diagnostics, Basel, Switzerland) by using 5 ng cDNA and 150 nM of each primer in a total volume of 10 µL containing SYBR Green PCR Master Mix (SYBR Green I Dye, AmpliTaq Gold DNA polymerase and dNTPs with dUTP instead of dTTP, Applied Biosystems, Thermofisher Scientific, Waltham, MA, USA), in accordance with the manufacturer’s instructions. Samples were normalized to hypoxanthine guanine phosphoribosyl transferase (HPRT) by calculating the ΔCt (Ct gene of interest—Ct HPRT); expression of the different genes is given as 2-(ΔCt). The qPCR was repeated twice for ALP, Col1A, CD44, TGF-β1, and ICAM-1 but only once for E-cad and BMP2. In Table 1, the sequences of the DNA primers used in the qPCR analysis are displayed.

### 2.4. Data Analysis

The primary outcomes of this in vitro study are the proliferation (viable cell/mL) of EPs, OBs, and PDLs and their relative gene expression for ALP, coll1A, CD44, TGF-β1, ICAM-1, E-cad, and BMP2 on day 3, comparing the groups of the experimental and control plates. The secondary outcome is the viability of cells on day 3. A Shapiro–Wilk normality test was performed to check the normality of the data. Data were not normally distributed; therefore, non-parametric tests were chosen. Data for proliferation and gene expression were reported as median in logarithmic scales or power of 10, while data for viability were reported in percentages. Multiple comparisons were performed for each cell type according to the medium and cell interplay for both the control and experimental models. For this purpose, a Kruskal–Wallis test with post hoc Dunn’s multiple comparisons test was used, while a Mann–Whitney test was performed for the group analysis. After the multiple comparison, for each analyzed cell type, data deriving from the experimental and control groups were clustered for the group analysis. The group analysis was performed in order to (a) increase the power, (b) investigate the effect of HA in its two forms used for this experiment, meaning HA diluted in medium (HAsol) versus HA for well coating (HAcoat), (c) the effect of both forms (soluble and coated) HA in the culture plates (+HA) compared to wells cultured without HA (−HA), and (d) the influence of culture wells without cell interaction (−comm) in contrast to those cell blocks simulating the communication between the cell types (+comm) on proliferation and relative gene expression on day 3. Data were analyzed and displayed through GraphPad 8.0 (GraphPad Software, San Diego, CA, USA). Differences were considered statistically significant for *p* < 0.05.

## 3. Results

### 3.1. Cell Viability

On day 3, the viabilities of the PDLs, OBs, and EPs were still high and comparable for both multi-culture cell plates and regular plates under all the incubations. No influence of the culture model, medium (with or without HA), or possible cell interaction on cell viability was observed. All cell types showed a median viability greater or at least 90%.

### 3.2. Proliferation of EPs, OBs, and PDLs

**Figure 2:**
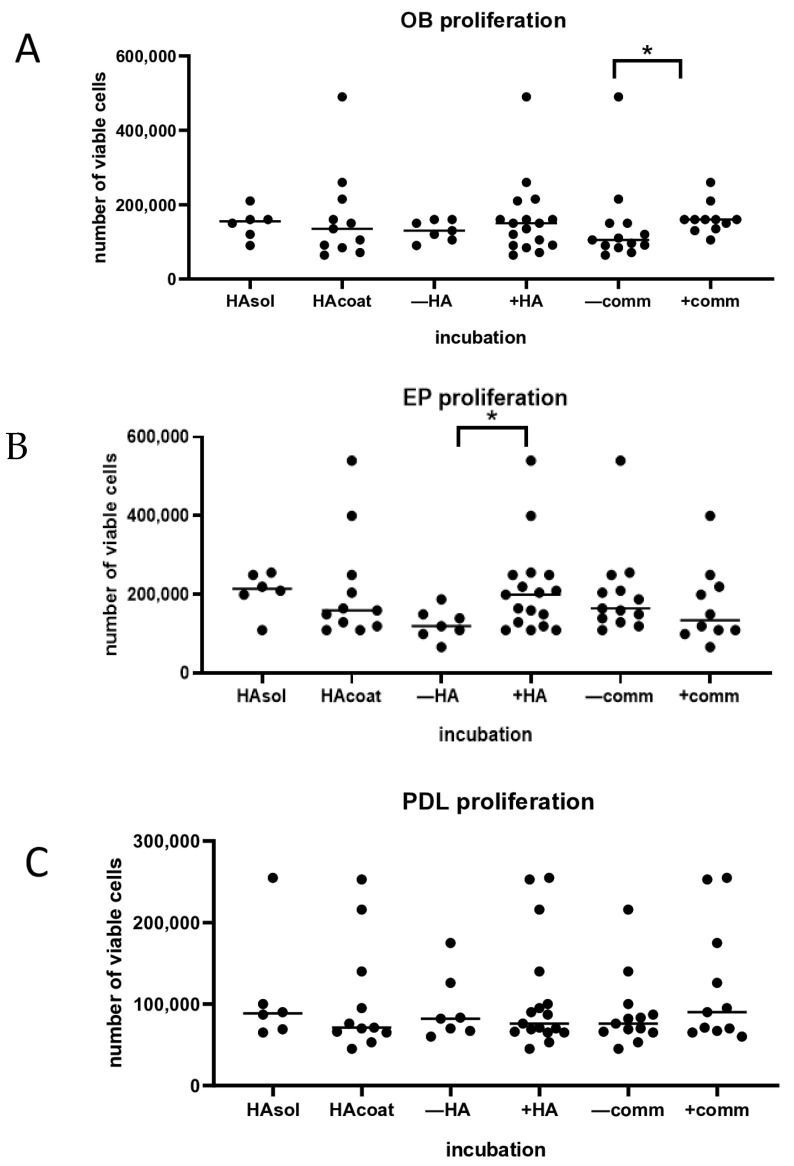
Cell proliferation (number of viable cell/mL) after 3 days of culturing with or without HA and with or without communication. (**A**) OBs (* *p* < 0.05); (**B**) EPs (* *p* < 0.05); (**C**) PDL cells. xHA = hyaluronic acid diluted in medium; HAcoat = hyaluronic acid coated on the plate; +HA = hyaluronic acid in medium; −HA = no hyaluronic acid in medium; −comm = culture plate without communication between the different cell types; +comm = cell-block plate with communication between the different cell types.

The three tested cell types showed proliferation compared to baseline number of cells per mL in the control and experimental plates on day 3. Nevertheless, the proliferation of EPs, OBs, and PDLs was independent from the type of culture plate, incubation, and cell communication. In the multiple comparison, the EP proliferation showed no significant difference among the experimental and control plate groups. Similarly to the EPs, the median proliferation of OBs and PDLs was not significantly different between the groups with soluble HA or coated with HA or with communication. Interestingly, in the group analysis, the proliferation of OBs was significantly higher after 3 days of culturing with cell communication than without cell communication (*p* < 0.05). Nevertheless, the proliferation of OBs was not influenced by the presence of HA in soluble or coated form (Figure 2A). EP proliferation was significantly increased by HA (*p* < 0.05) but not by cell interaction or the formulation of HA in the wells (Figure 2B). No differences were found in proliferation for the PDL cells (Figure 2C) in the group analysis.

### 3.3. Quantitative Polymerase Chain Reaction (qPCR)

**Figure 3:**
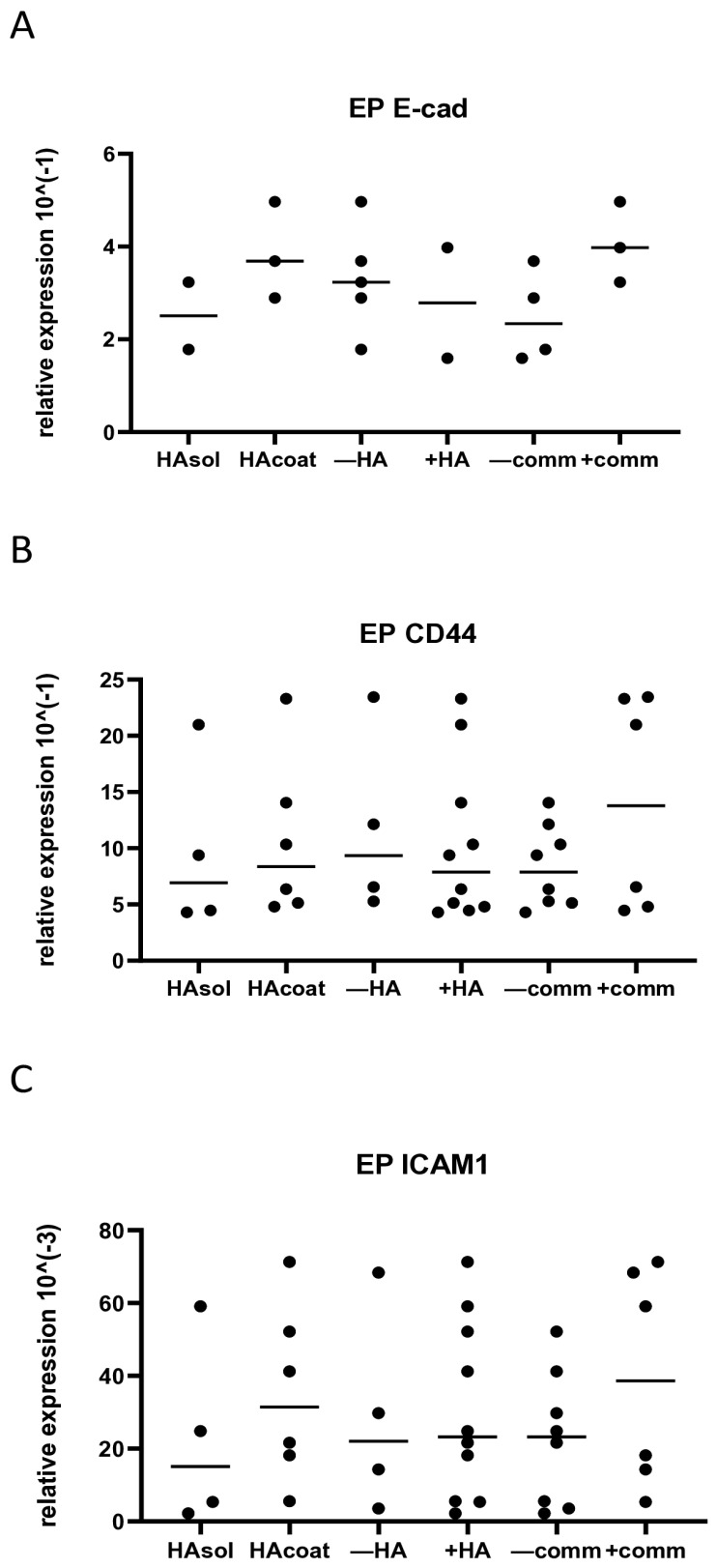
Relative gene expression of EPs after 3 days of culturing with or without HA and with or without communication. (**A**) E-cad expression; (**B**) CD44 expression; (**C**) ICAM1 expression. HAsol = hyaluronic acid diluted in medium; HAcoat = hyaluronic acid coated on the plate; +HA = hyaluronic acid in soluble and coated form; −HA = no hyaluronic acid in medium; −comm = culture plate without communication between the different cell types; +comm = cell-block plate with communication between the different cell types.

**Figure 4:**
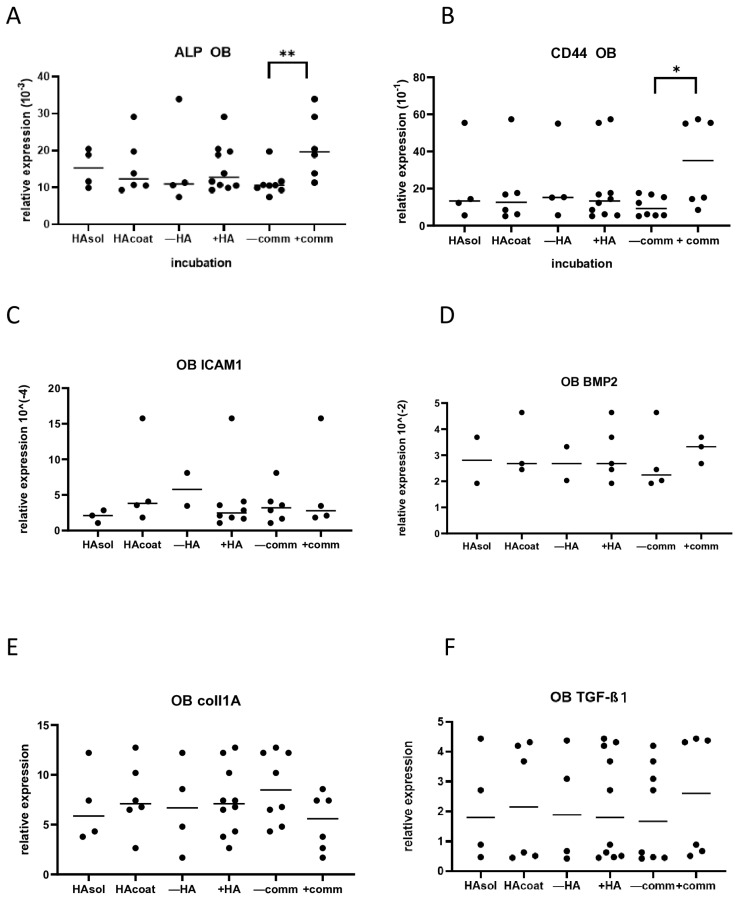
Relative gene expression of OBs after 3 days of culturing with or without HA and with or without communication. (**A**) ALP expression (** *p* < 0.01); (**B**) CD44 expression (* *p* = 0.08); (**C**) ICAM1 expression; (**D**) BMP2 expression; (**E**) Coll1A expression; (**F**) TGF-β1 expression. HAsol = hyaluronic acid diluted in medium; HAcoat = hyaluronic acid coated on the plate; +HA = hyaluronic acid in soluble and coated form; −HA = no hyaluronic acid in medium; −comm = culture plate without communication between the different cell types; +comm = cell-block plate with communication between the different cell types.

**Figure 5:**
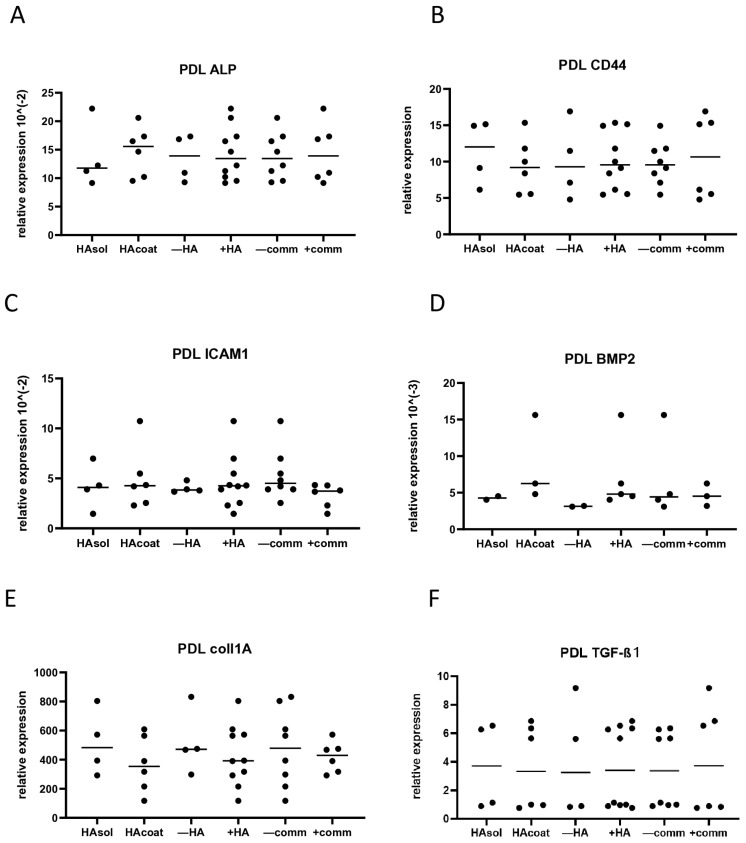
Relative gene expression of PDLs after 3 days of culturing with or without HA and with or without communication. (**A**) ALP expression; (**B**) CD44 expression; (**C**) ICAM1 expression; (**D**) BMP2 expression; (**E**) Coll1A expression; (**F**) TGF-β1 expression. HAsol = hyaluronic acid diluted in medium; HAcoat = hyaluronic acid coated on the plate; +HA = hyaluronic acid in soluble and coated form; −HA = no hyaluronic acid in medium; −comm = culture plate without communication between the different cell types; +comm = cell-block plate with communication between the different cell types.

After 3 days of culturing EPs, OBs, and PDLs, cells were lysed for gene expression. The expression was measured for E-cad, BMP2, ALP, CD44, ICAM-1, coll1A, and TGF-β1. Nevertheless, not all cell types expressed all investigated genes. For instance, E-cad expression was reported only for EPs since OBs and PDLs did not express it, while BMP2, ALP, Coll1A, and TGF-β1 were detected only in OBs and PDLs. In EPs, the median relative gene expression of E-cad, CD44, and ICAM1 demonstrated no significant variation between the groups (Figure 3A–C). In OBs, cell interaction significantly increased ALP expression (*p* < 0.01) and promoted a trend of higher CD44 expression (*p* = 0.08) (Figure 4A,B). All other tested genes did not display any differences when the cells were cultured with or without HA, or with or without communication (Figure 4C–F). PDL cells exhibited no significant difference in expression for any tested gene (Figure 5A–F).

## 4. Discussion

**Figure 6:**
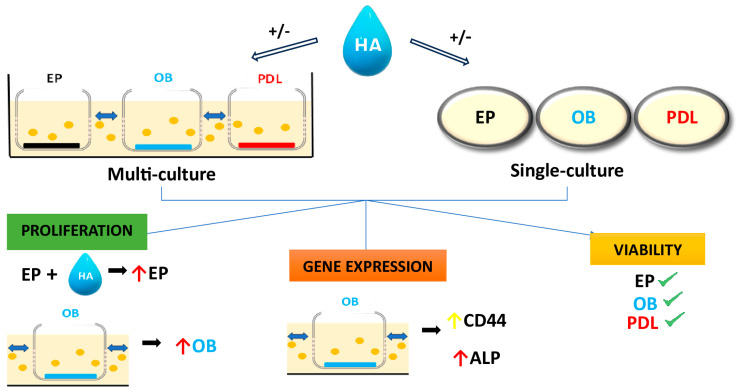
Periodontal ligament fibroblasts (PDL), osteoblasts (OB) and gingiva epithelial cells (EP) were cultured in a multi-culture plate and in a single-culture plate, with and without hyaluronic acid (HA). The multi-culture plate used in this experiment is designed to mimic the cell-cell interaction closer to the clinical situation where cells can interact with each other. The proliferation of EPs was significantly boosted by HA while OBs showed significant proliferation in case of intercellular communication in the multi-culture plate. A significantly higher gene expression of ALP and a trend of higher CD44 expression were detected in OBs in communication with the other cell types. The viability of all cell types was high and comparable in both plates after three days.

This in vitro study investigated the viability, proliferation, and gene expression of gingival epithelial cells, osteoblasts, and periodontal ligament fibroblasts when stimulated, for 3 days, with high-molecular-weight cross-linked HA and when these cells interact with each other, mimicking a “real clinical condition”. Several studies assessed the cell behavior in a co-culture model, improving the understanding of the cell-to-cell interplay mechanisms [32,33,34]. However, this is the first attempt at investigating the effect of HA on different periodontal cell types when these communicate by sharing the same medium. The choice of using cross-linked HA in the present study is based on the fact that both the non-cross-linked and cross-linked formulations promote the viability and cell proliferation of oral cavity cells, as already proven in previous studies [26,27]. The cross-linked formulation has the advantage of slowing down the degradation rate of HA by hyaluronidase, making it more favorable for clinical applications. Since HA gel is a highly hydrophilic material, it tends to dissolve in contact with body fluids. Therefore, in this experiment, we opted to assess HA when diluted in a medium but also when applied for well coating. The results demonstrate that the use of HA, independently from its formulation, can keep the viability of cells high for at least 3 days. This could be explained by the exceptional ability of HA to bind to water molecules, which helps to keep cells hydrated [35]. Another remarkable finding related to the cell viability is that the novel multi-culture plate seems to be highly biocompatible with periodontal cells. This suggests its valid use when multiple communicating periodontal cells are analyzed in an in vitro setting. Regarding the proliferation of cells among the different experimental and control groups, the limited number of samples may explain the inconsistency of the results in the multiple comparison. To compensate the low number of samples, but also for a better understanding of the cell behavior in the different tested conditions, we opted to cluster the samples in six “macrogroups” in order to have three comparisons. In the first comparison, cells were analyzed when in contact with the two formulations of HA (HA diluted in medium, “HAsol”, vs. HA for well coating, “HAcoat”). In the second comparison, cells were stimulated with (soluble and coated) or without HA (“+HA” vs. “−HA”). In the third comparison, we examined the effect of communicating wells and non-communicating wells (“−comm” vs. “+comm”) on the cells. The group analysis revealed that the use of HA can significantly boost the number of epithelial cells after 3 days compared to the group without HA. For EP proliferation, the communication factor appeared to be not relevant. In the literature [36], HA has been shown to have various effects on EPs, including the promotion of cell proliferation and migration as well as stimulation of wound healing. A hyaluronan-rich matrix can be associated with proliferating basal keratinocytes and facilitates keratinocyte migration through CD44-mediated mechanisms [19,34,35,36,37]. CD44 is the predominant receptor of HA in most cells [19], and this was also ascertained in our study. On the basis of our findings, we hypothesize that HA may be advantageous in all those surgical procedures where rapid epithelialization or wound closure is required [38]. Nevertheless, in the current in vitro study, the significantly higher number of EPs was not accompanied by an amplified expression of E-cad, which is crucial for epithelial cell–cell adhesion [39]. Contrarily to EP proliferation, cell communication, independently from HA, increased the proliferation and gene expression of ALP and CD44 for OBs. The latter indicates a potential osteogenic stimulation (Figure 6). HA is able to bind selectively to CD44, and this determines the activation of an intracellular signaling cascade which regulates cell proliferation, differentiation, and survival [19,40]. Despite the promising results deriving from the literature, the osteogenic potential of HA remains a controversial topic [14,27,31]. Previous in vitro studies were not designed with different cell types communicating with each other, while in the current experiment, we assessed cells in a culture environment that better mimics an in vivo situation. Differently from our investigation, in the study by Asparuhova et al. [14], after 24 h starvation, the proliferative rate of an osteoprogenitor cell line (MC3T3-E1) treated with 4 mg/mL diluted cross-linked HA was enhanced by 11.7-fold compared to control cells. The different results between the above study and the present study could be explained by the lack of starvation in our experiment, different dilution factors, and different cell lines. In addition, HA significantly inhibited BMP2 expression, corroborating the results reported by Kaneko et al. [41], suggesting that HA may inhibit BMP-induced osteoblastic differentiation through the CD44 receptor. Different findings were also shown in the study by Zhao et al. [10], in which high-molecular-weight HA and high concentrations increased the mRNA expressions of ALP, RUNX-2, and OCN rather than cell adhesion and proliferation, while low-molecular-weight HA displayed stimulation of proliferation. In the present study, the significant OB proliferation may be associated with the not significant but greater trend of CD44 mRNA expression when OBs were allowed to be in communication with EPs and PDLs. We speculate that OB proliferation and activity could directly or indirectly have been triggered by exosomes or extracellular vesicles present in the interconnected medium. Exosomes are nanovesicles of 30–150 nm in diameter that are secreted by a large variety of cells [42]. These extracellular vesicles are specialized in multiple functions and could promote the osteogenic differentiation of periodontal ligament stem cells [43,44,45]. However, further research is needed to fully understand the mechanisms underlying their actions. Our findings also revealed that ALP gene expression was higher in OBs cultured in communication with the other two cell types. ALP is a membrane-bound glycoprotein well known as an early osteogenic marker of bone formation and bone calcification [46]. It is remarkable to note that, in the current study, HA or cell interplay had no effects on the proliferation and function of PDL cells, and we currently cannot explain this. These inconclusive outcomes deriving from PDLs may be related to the short observation time, no starvation of the cells prior the experiment, no biochemical cell counting system, and a lower number of cells for the real-time PCR in our study compared to other in vitro studies [14,26]. The present experiment model shows some limitations: the in vitro study was designed to last a short time (three days). Consequently, its effect on periodontal cells may be different for longer observations. Another drawback is that the results are influenced by the limited number of analyzed samples. This inevitably affected the statistical robustness, underestimating possible relevant differences among the groups. Finally, considering the alignment of EP-OB-PDL in the experimental plate, OBs may have benefitted more from the “communication” compared to the relatively more peripheral position of PDLs and EPs. In terms of future perspective, this study may represent a new direction in evaluating biomaterials for periodontal applications. On the basis of our findings, future studies unraveling the biological pathways through which HA influences cell proliferation and osteogenesis are recommended. The emphasis on the development of dynamic and complex in vitro models may be necessary to mimic the physiological conditions of the periodontium and provide insights into the dynamic interactions among cells and biomaterials or when different biomaterials are compared. In particular, if the role of HA in promoting osteogenesis and stimulating PDLs can be further elucidated in multi-culture models, this could set new treatment strategies for periodontal disease, especially in cases requiring tissue regeneration.

## 5. Conclusions

HA seems to have the capacity to induce EP proliferation, while the communication among different periodontal cells may be associated with osteogenic stimulation. The use of multi-culture plates could be a promising starting point for more closely mimicking the in vivo environment to examine the effect of dental biomaterials on various cell types of the periodontium. The potential role of HA in periodontal regeneration needs further mechanistic studies in complex and dynamic environments to support its clinical use.

## Figures and Tables

**Table 1:** Primer sequences used for quantitative real-time PCR analysis.

Genes	Primer Sequence (5′–3′)
ALP	FORWARD: gCTTCAAACCgAgATACAAgCA REVERSE: gCTCgAAgAgACCCAATAggTAgT
coll1A	FORWARD: TCCAACgAgATCgAgATCC REVERSE: AAgCCgAATTCCTggTCT
E-Cad	FORWARD: TACgCCTgggACTCCACCTA REVERSE: CCAgAAACggAggCCTgAT
BMP-2	FORWARD: gCCAgCCgAgCCAACAC REVERSE: AAATTAAAgAAgAATCTCCgggTTgT
TGF-β1	FORWARD: CACCCgCgTgCTAATggT REVERSE: CTCggAgCTCTgATgTgTTgAA
ICAM-1	FORWARD: TgAgCAATgTgCAAgAAgATAgC REVERSE: CCCgTTCTggAgTCCAgTACA
CD44	FORWARD: TggCTgATCATCTTggCATCC REVERSE: TTgCTggCCTCTCCgTTgAgT
HPRT	FORWARD: TgACCTTgATTTATTTTgCATACC REVERSE: CgAgCAAgACgTTCAgTCCT

## Data Availability

The original contributions presented in this study are included in the article. Further inquiries can be directed to the corresponding authors.
